# BIRTHWEIGHT, LIFETIME OBESITY, AND PHYSICAL FUNCTIONING IN MID-ADULTHOOD: A NATIONWIDE BIRTH COHORT STUDY

**DOI:** 10.1093/geroni/igz038.190

**Published:** 2019-11-08

**Authors:** Nina Rogers

**Affiliations:** University College London, London, United Kingdom

## Abstract

Evidence is scant on long-term implications of body mass index (BMI) gains over the life-course for poor physical functioning (PF). Using the 1958 British birth cohort (N=8,674) we examine whether i) birthweight and BMI across the life-course; ii) BMI gains at specific life-stages; and iii) age of obesity onset, were associated with PF at 50y. At each adult age, obesity was associated with poor PF (e.g. for males at 23y adjusted-ORs for poor PF was 2.28(1.34,3.91)). BMI gains were associated with poor PF (e.g. for females, adjusted-OR per SD BMI gain 16-23y was 1.28(1.13,1.46)). Longer obesity duration was associated with poor PF (e.g. for males, adjusted-OR was 2.32(1.26,4.29) for childhood obesity onset, and 1.50(1.16,1.96) for mid-adulthood onset); associations were abolished with further adjustment for 50y BMI. Obesity, BMI gains, and earlier obesity onset were associated with poor PF in mid-adulthood reinforcing the importance of preventing obesity early in the lifecourse.

